# Dose distribution and tumor control probability in out-of-field lymph node stations in intensity modulated radiotherapy (IMRT) vs 3D-conformal radiotherapy (3D-CRT) of non-small-cell lung cancer: an *in silico* analysis

**DOI:** 10.1186/s13014-015-0485-6

**Published:** 2015-08-21

**Authors:** Jochen Fleckenstein, Andrea Eschler, Katharina Kremp, Stephanie Kremp, Christian Rübe

**Affiliations:** Department of Radiotherapy and Radiation Oncology, Saarland University Medical School, 66421 Homburg, Germany; Department of Diagnostic and Interventional Radiology, Saarland University Medical School, Homburg, Germany

## Abstract

**Background:**

The advent of IMRT and image-guided radiotherapy (IGRT) in combination with involved-field radiotherapy (IF-RT) in inoperable non-small-cell lung cancer results in a decreased incidental dose deposition in elective nodal stations. While incidental nodal irradiation is considered a relevant by-product of 3D-CRT to control microscopic disease this planning study analyzed the impact of IMRT on dosimetric parameters and tumor control probabilities (TCP) in elective nodal stations in direct comparison with 3D-CRT.

**Methods and materials:**

The retrospective planning study was performed on 41 patients with NSCLC (stages II-III). The CTV was defined as the primary tumor (GTV + 3 mm) and all FDG-PET-positive lymph node stations. As to the PTV (CTV + 7 mm), both an IMRT plan and a 3D-CRT plan were established. Plans were escalated until the pre-defined dose-constraints of normal tissues (spinal cord, lung, esophagus and heart) were reached. Additionally, IMRT plans were normalized to the total dose of the corresponding 3D-CRT. For two groups of out-of-field mediastinal node stations (all lymph node stations not included in the CTV (LN_all_el_) and those directly adjacent to the CTV (LN_adj_el_)) the equivalent uniform dose (EUD) and the TCP (for microscopic disease a D50 of 36.5 Gy was assumed) for the treatment with IMRT vs 3D-CRT were calculated.

**Results:**

In comparison, a significantly higher total dose for the PTV could be achieved with the IMRT planning as opposed to conventional 3D-CRT planning (74.3 Gy vs 70.1 Gy; *p* = 0.03). In identical total reference doses, the EUD of LN_adj_el_ is significantly lower with IMRT than with 3D-CRT (40.4 Gy vs. 44.2 Gy. *P* = 0.05) and a significant reduction of TCP with IMRT vs 3D-CRT was demonstrated for LN_all_el_ and LN_adj_el_ (12.6 % vs. 14.8 %; and 23.6 % vs 27.3 %, respectively).

**Conclusions:**

In comparison with 3D-CRT, IMRT comes along with a decreased EUD in out-of-field lymph node stations. This translates into a statistically significant decrease in TCP-values. Yet, the combination of IF-RT and IMRT leads to a significantly better sparing of normal tissues and higher total doses whereas the potential therapeutic drawback of decreased incidental irradiation of elective lymph nodes is moderate.

## Background

Radiochemotherapy is the standard treatment for inoperable, non-metastasized non-small-cell lung cancer (NSCLC). Radiotherapy planning of NSCLC underwent major changes during the past decade. Both the integration of FDG-positron emission tomography (FDG-PET) and the shift to involved-field radiotherapy (IF-RT) became a new therapeutic standard [1]. It was shown in a series of trials that IF-RT (in the context of FDG-PET based treatment planning) does not go along with an unacceptable risk of isolated failure in ‘out-of-field’ lymph node stations but allows for further dose escalation [2–6]. The favorable low rates of ‘out-of-field’-failure with IF-RT were also attributed to a significant dose-deposition in elective nodal areas with the use of 3D-conformal radiotherapy (3D-CRT) [7, 8]. Meanwhile, a transition from 3D-CRT to intensity modulated radiotherapy (IMRT) in NSCLC takes place [9, 10]. Given the fact that IMRT is of additional benefit with respect to dose escalation and the sparing of relevant organs at risk (OAR) [11–13], it is yet unclear if the high dose conformality of IMRT implies an adverse effect on tumor control probability (TCP) for microscopic disease in the “rind”-region outside the clinical target volume. This therapeutic backdrop was the incentive to perform the presented *in silico*-analysis, which is a head-to-head comparison of IMRT and 3D-CRT in terms of dose distribution and hypothetical TCP in out-of-field mediastinal and hilar lymph node stations. It was carried out on a group of 41 patients who had participated in the ‘*PET-PLAN* pilot trial’, which examined the rate of isolated ‘out-of-field’ nodal failures with FDG-PET based IF-RT [6].

## Methods

### Patient selection and planning conditions

Forty-one patients who had been enrolled in the previously published ‘*PET-PLAN* pilot trial’ were selected for the presented *in silico* analysis [6]. It was approved by the local ethics committee (*Ärztekammer des Saarlandes*), and all participating patients had given written informed consent. All selected patients had pathologically confirmed, medically inoperable stage II-III NSCLC and were candidates for definitive radio(chemo)therapy. Further inclusion criteria and the detailed modality of both the computed tomography and ^18^F-FDG-PET acquisition as prerequisites for the treatment planning are described in the preceding publication [6].

### Target volumes and treatment planning (IMRT and 3D-CRT)

FDG-PET-based target volumes were defined by a radiation oncologist with the support of a radiation physicist and a nuclear medicine physician. The registration of CT and FDG-PET-image sets was scrutinized for anatomical plausibility and – if necessary - manually adjusted. The gross tumor volume (GTV) of the primary tumor was initially autocontoured with a contrast-oriented source-to- background (‘S/B’) algorithm for FDG-PET–based delineation of tumor volumes as described by Nestle et al. [14] and subsequently adjusted to the tumor borders as shown in the CT scan displayed in soft tissue and lung window. FDG-PET–negative atelectasis was excluded from the GTV. The GTV was expanded to the clinical target volume (CTV) by 3 mm in all dimensions; then, the CTV was expanded by another 7 mm to create the planning target volume (PTV) for the primary tumor.

As to the involved lymph nodes, only American Joint Committee of Cancer (AJCC)-lymph node stations containing FDG-PET-positive lymph nodes were contoured as CTV, referring to an anatomical contouring guide [15], whereas CT-positive but FDG-PET-negative lymph nodes were disregarded. The nodal CTV was then expanded by 7 mm to obtain the PTV for the involved lymph node stations. Finally, the definitive PTV was obtained from the unification of the PTVs of the primary tumor and the involved lymph node stations.

Dose constraints for normal tissues were defined as follows: for the *whole lung* (the GTV of primary tumor had been subtracted from the contoured lung volume) V_20_ ≤ 35 %, and mean lung dose (MLD) ≤ 20 Gy; the *spinal cord* 45 Gy as maximum; the *esophagus* V_50_ ≤ 50 % or mean dose ≤34 Gy and maximum dose ≤70 Gy; and the *heart* V_45_ ≤ 67 % and V_60_ ≤ 33 %.

For each patient, both an IMRT- and a 3D-CRT-treatment plan were generated and optimized by an experienced radiation physicist, using the PINNACLE^3^ treatment planning system (Version 8.2, Philips Medical Systems, Best, The Netherlands). IMRT “step-and shoot” plans (photon energy: 6 MV) were calculated using ‘direct machine parameter optimization’. The final dose distribution was calculated with a collapsed cone algorithm. Typically, six or seven coplanar beams with 70 segments were used; the beam angles were individually adapted. Inverse planning was started with a default prescription dose of 70 Gy and standardized objectives for the coverage of the planning target volume (PTV) as provided by the ICRU Report 83 were applied [16]. To allow for potential dose escalation, pre-defined lower OAR constraints were used as default values for the initial 70 Gy plan: whole lung – V20 < 28 %; heart – V40 < 20 %; spinal cord – maximum dose of 40 Gy; esophagus – V50 < 50 % and maximum dose of 70 Gy. The total prescription dose was escalated or deescalated by 2 Gy starting with 70 Gy until all dosimetric premises with respect to PTV coverage and OARs were met. To compare IMRT with 3D-CRT plans at equal dose levels with respect to dose distribution in out-of-field lymph node stations and sparing of OARs, IMRT plans were additionaly normalized to the same maximum dose level (defined as total reference dose, D_ref_) as it was reached with 3D-CRT plans (adapted doses are labeled as IMRT_norm_). The normalization was achieved by matching the number of fractions of the IMRT plans with those of the 3D-CRT plans (no *de novo* IMRT-planning was performed for dose normalization).

For 3D-CRT planning at least three beams had to be used and the number and arrangement of beams and their weights, the use of wedges as well as the photon energy (6 and 18 MV) were optimized by an experienced medical physicist.

A minimal total prescription dose of 60 Gy was aspired and depended on the dose constraints for normal tissues. The prescribed single dose was 2 Gy and the total prescription dose was incrementally escalated by 2 Gy as long as the dose constraints outlined above were not surpassed. The maximum doses for both the IMRT and the 3D-CRT plan were limited to 110 Gy.

### Contouring and grouping of out-of-field lymph node stations

All hilar and mediastinal AJCC-lymph node stations were contoured based on the individual CT-anatomy according to the atlas provided by Chapet et al. [15]. Specifically, two nodal ‘out-of-field’-volumes were defined for each patient. The first volume, LN_all_el_, comprised the entire elective hilar and mediastinal lymph node stations, i.e. all uninvolved levels outside the CTV. The second volume, LN_adj_el_, included only the elective ‘out-of-field’-lymph node levels 7 (infracarinal, if uninvolved), the ipsilateral hilum (levels 10/11, if uninvolved) and those uninvolved and directly adjacent to (involved) lymph node levels included in the CTV. For example, if only level 4R (deep paratracheal lymph nodes, right side) was involved, the volume Ln_adj_el_ contained the bordering lymph node levels 2R, 4 L, 7 and 10_11R.

### Dose distribution in out-of-field nodal regions

Dose volume histograms (DVH) were created for both delineated out-of-field lymph node volumes (LN_all_el_, LN_adj_el_) for three plans in each patient (3D-CRT, IMRT and IMRT_norm_). For LN_all_el_ and LN_adj_el_ mean doses were calculated and additionally (in order to correct for heterogeneity in dose distribution), the equivalent uniform dose (EUD) was derived from DVH-based values by using the formula as given by Wu et al. [17]:

$$ EUD={\left(\frac{1}{N}{\displaystyle {\sum}_i{D}_i^a}\right)}^{\frac{1}{a}}, $$

where N is the number of voxels in the anatomic region of interest, *D*_*i*_ the dose in the *i* th voxel, and *a* is a tumor-dependent value, which describes the dose-volume effect. The EUD-concept was designed to translate a heterogeneous dose distribution in a given volume into an isoeffective (hypothetical) homogeneous dose distribution with the compared biologic effect mostly being tumor control. One advantage of the EUD-concept is that it simplifies the comparison of volumes with various heterogeneous dose-distribution while it remains at the same time an objective measure.

### Tumor control probability

TCP values were computed for LN_all_el_ and LN_adj_el_ in all three plan variants (3D-CRT, IMRT and IMRT_norm_). For TCP calculation, the formula given by Martel et al. was applied [18]:

$$ TCP=\frac{1}{1+{\left(\frac{D_{50}}{D}\right)}^{4\gamma }}, $$

where D_50_ is the dose needed to obtain a 50 % TCP, D is the actual total dose deposited in the irradiated volume, and γ is the normalized slope of the sigmoidal response curve at D_50_. Parameters to control microscopic disease in lung cancer were used as provided by Okunieff et al. [19]. Precisely, D was derived from the computed EUD, D_50_ was 36.5 Gy and γ was 0.72.

### Statistical analysis

The paired sample *t-*test was used to test for statistical differences between data sets in case of normal distribution, otherwise the Wilcoxon signed rank test was applied. Differences were considered significant when the *p-*value was <0.05. Origin Pro 9.0 (OriginLab Corporation, Northampton, MA) was used for the statistical analysis.

## Results

### Study population

The patient characteristics are presented in Table 1. Primary tumors were located as follows: centrally (left: *n* = 10; right: *n* = 16), left upper lobe (*n* = 6), left lower lobe (*n* = 3), right upper lobe (*n* = 3) and right lower lobe (*n* = 3). Thirty-nine patients had lymph node involvment as diagnosed with FDG-PET. Six patients had a ‘single level’ involvement and 33 patients had a ‘multilevel’ spread. The mean number of involved lymph node stations was 2.5 ± 0.9 sd (standard deviation).

**Table 1:** Patient characteristics

Total no. of patients	41
Age, years	
Mean ± SD	72,5 ± 7,5
Range	56–87
Sex, no. (%)	
Male	37 (90)
Female	4 (10)
KPS, no. (%)	
70	9 (22)
80	17 (41)
90	15 (37)
100	0
T-Stage, no. (%)	
T1	2 (5)
T2	9 (22)
T3	13 (32)
T4	17 (41)
N-Stage^a^, no. (%)	
N0	2 (5)
N1	5 (12)
N2	31 (76)
N3	3 (7)
UICC stage, no. (%)	
IIB	3 (7)
IIIA	17 (42)
IIIB	21 (51)
FEV1, % predicted	
Mean ± SD	63 ± 15

*SD* standard deviation, *KPS* Karnofsky Performance Score, *FEV1* Forced expiratory volume during 1^st^ second of breathing maneuver

^a^As staged with FDG-PET

### Comparison of IMRT and 3D-CRT planning

**Fig. 1:**
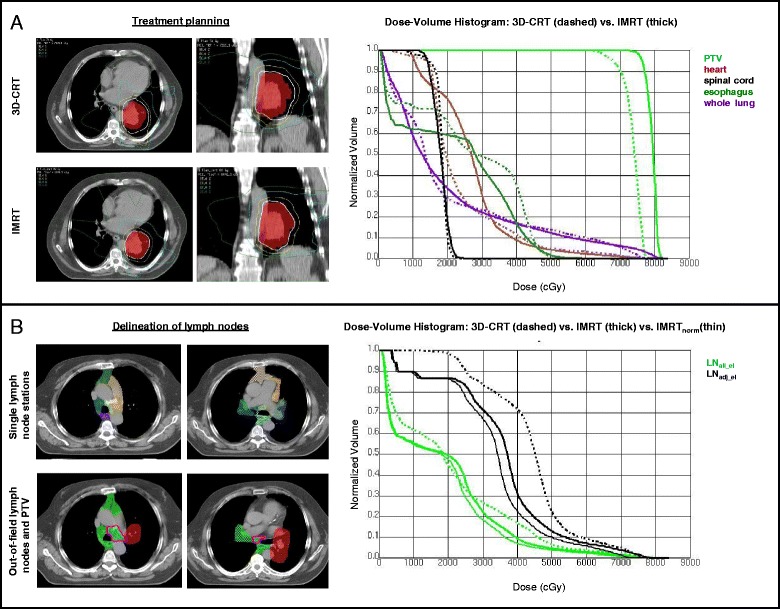
Comparison of 3D-CRT vs. IMRT-treatment planning, shown for one patient with stage IIIA NSCLC in the left hilum. **a** IF-RT planning with treatment volume: FDG-PET based PTV (*red, colorwash*). With IMRT planning (*second row*) administration of a total dose of 78 Gy would be possible vs. 72 Gy with 3D-CRT (first row). Mean lung dose was dose limiting both in 3D-CRT and IMRT (see dose-volume-histogram (DVH)). **b** Example for atlas-based individual delineation of single thoracic lymph node stations (*first row*) according to Chapet et al. [15]. Out-of-field lymph node stations were subsequently merged to two regions of interest: first, LN_all_el_, i.e. all out-of-field lymph nodes stations (*green, colorwash*), and second, LN_adj_el_, i.e. uninvolved lymph node stations 7 and 10/11 (ipsilateral) or anatomically adjacent to involved lymph node stations (*pink line*). The DVH reveals a lower dose exposition for both sets of out-of-field lymph node stations for two IMRT-plans (with or without additional dose escalation (IMRT and IMRT_norm_, respectively) as opposed to 3D-CRT

Relevant PTV-based planning parameters are shown in Table 2. With IMRT-planning a significangly higher mean total dose could be achieved as compared with 3D-CRT (74.3 vs. 70.1 Gy, *p* < 0.00001). Also, 7 of 41 patients could be administered a total dose of 66 Gy or more with IMRT. These results can be ascribed to the superior dose conformality of IMRT vs. 3D-CRT (CI: 0.79 vs. 0.50, *p* < 0.00001) resulting in an improved sparing of OAR, which is shown in Table 3. At normalized dose levels, IMRT was superior to 3D-CRT in sparing the spinal cord (Dmax) and limiting the exposure of the esophagus with higher doses (V_60_). Differences between IMRT and 3D-CRT in regard to PTV coverage and OAR-exposure are exemplified in one patient in Fig. 1a.

**Table 2:** IMRT vs. 3D-CRT: PTV based dosimetric parameters of all 41 patients

	IMRT	3D-CRT	p
PTV, cm^3^	433.3 ± 168.8		
Total dose (D_ref_)			
Median (range), Gy	72 (62–110)	70 (58–100)	
Mean ± SD, Gy	74.3 ± 9.1	70.1 ± 7.9	<0.00001
≥60 Gy, no. (%)	41 (100)	40 (98)	
≥66 Gy, no. (%)	39 (95)	32 (78)	
D_mean_-PTV, Gy ± SD	73.6 ± 9.0	70.0 ± 8.1	<0.00001
D_90_-PTV, Gy ± SD	71.3 ± 9.0	66.9 ± 7.8	<0.00001
CI ± SD	0.79 ± 0.04	0.50 ± 0.10	<0.00001

*IMRT* intensity modulated radiotherapy, *3D-CRT* 3D-conformal radiotherapy, *PTV* planning target volume, *D*_*ref*_ prescribed reference dose, *SD* standard deviation, *D*_*mean*_ mean dose in predefined region of interest, *D*_*90*_ dose, administered to 90 % of region of interest, *CI* conformal index

**Table 3:** IMRT vs. 3D-CRT: dosimetric parameters for relevant organs at risk of all 41 patients. IMRT dose was also normalized to a prescription dose equaling 3D-CRT dose in each patient, indicated as IMRT_norm_

	IMRT	IMRT_norm_	3D-CRT	p
Lung				
mean dose, Gy	17.3 ± 2.7	16.7 ± 2.7	17.2 ± 3.0	
V_20_, %	28.3 ± 4.9	27.2 ± 7.3	28.4 ± 5.3	
Esophagus				
mean dose, Gy	26.8 ± 9.0	25.3 ± 9.3	26.0 ± 10.0	
V_60_, %	14.8 ± 12.8	11.1 ± 12.2	17.1 ± 14.4	<0.0001*
V_50_, %	25.7 ± 18.1	23.0 ± 17.4	29.1 ± 18.5	
D_max_, Gy	65.7 ± 8.6	59.6 ± 13.9	63.6 ± 12.0	<0.05*
Spinal cord				
D_max_, Gy	38.8 ± 6.9	36.3 ± 7.4	39.5 ± 9.7	<0.01*
Heart				
V_60_, %	2.1 ± 2.6	1.5 ± 1.9	4.6 ± 5.8	<0.01*
V_45_, %	6.7 ± 8.6	4.8 ± 5.9	10.4 ± 11.3	<0.01*

Data are presented as means ± standard deviation

*IMRT* intensity modulated radiotherapy, *3D-CRT* 3D-conformal radiotherapy, *D*_*max*_ maximum point dose within the defined organ at risk, *V*_*20*_, *V*_*10*_ … percentage of volume of the defined organ at risk receiving more than the indicated dose

*Test for statistical significance performed for IMRT_norm_ vs. 3D-CRT (values only indicated if significant)

### Dose distribution and TCP in out-of-field lymph node stations

Values referring to mean dose, EUD, and TCP in LN_all_el_ and LN_adj_el_ are presented in Table 4. If regarded at normalized dose levels (IMRT_norm_ vs. 3D-CRT), mean dose in LN_all_el_ and LN_adj_el_ was significantly decreased with IMRT_norm_. EUD was significantly decreased with IMRT and IMRT_norm_ in comparison with 3D-CRT. The significantly lower doses in LN_all_el_ and LN_adj_el_ with IMRT_norm_ vs. 3D-CRT translated into significantly, but overall moderately reduced values in TCP. TCP-values did not differ significantly between IMRT and 3D-CRT if IMRT is used for additional dose-escalation.

**Table 4:** IMRT vs. 3D-CRT: PTV based dosimetric parameters stratified by treatment volume concept for all 41 patients. IMRT dose was also normalized to a prescription dose equaling 3D-CRT dose in each patient, indicated as IMRT_norm_

	IMRT	IMRT_norm_	3D-CRT	p
Mean dose (Gy)				
LN_all_	36.7 ± 11.0	32.5 ± 7.5	37.1 ± 9.9	<0.0001*
LN_adj_	46.6 ± 9.7	37.9 ± 9.3	47.6 ± 8.6	<0.0001*
EUD (Gy)				
LN_all_	32.2 ± 8.7	30.5 ± 8.3	33.5 ± 9.7	<0.01**/<0.00001*
LN_adj_	42.7 ± 9.3	40.4 ± 8.7	44.2 ± 9.0	<0.001**/<0.00001*
TCP (%)				
LN_all_	13.8 ± 11.2	12.6 ± 9.9	14.8 ± 13.2	<0.01*
LN_adj_	25.7 ± 21.4	23.6 ± 19.4	27.3 ± 21.7	<0.001*

Data are presented as means ± standard deviation

*IMRT* intensity modulated radiotherapy, *3D-CRT* 3D-conformal radiotherapy, *PTV* planning target volume, *LN*_*all*_ all hilar and mediastinal lymph node stations without evidence of disease as staged with FDG-PET, *LN*_*adj*_ hilar and mediastinal lymph node stations without evidence of disease as staged with FDG-PET, but directly adjacent to involved lymph node stations, *TCP* tumor control probability, *ns* not significant

*Test for statistical significance performed for IMRT_norm_ vs. 3D-CRT (values only indicated if significant)

**Test for statistical significance performed for IMRT vs. 3D-CRT (values only indicated if significant)

In Fig. 1b the delineated out-of-field lymph node stations and the resulting DVH for both volumes LN_all_el_ and LN_adj_el_ are depicted for one patient.

## Discussion

The presented planning study revealed a potential hazard in the use of IMRT in IF-RT of locally advanced NSCLC: IMRT goes along with a decreased dose deposition in out-of-field lymph node stations and may thus involve the risk of an increased rate of out-of-field nodal failure. However, this holds true only under the condition that 3D-CRT and IMRT are compared at equalized dose levels. Furthermore, the calculated differences in TCP were slim between IMRT_norm_ and 3D-CRT (12.6 % vs. 14.8 %). As our results show: if the full potential of IMRT for dose escalation were exploited, no signficant difference would be observed in TCP-values compared to 3D-CRT, while EUD in out-of-field lymph node stations still remained lower. These findings indicate that one cannot count as much on IMRT as on 3D-CRT to compensate for inaccurate nodal staging by providing effective incidental dose-coverage in elective nodal regions. It is of note that only an increased rate of isolated nodal failure (as opposed to a combined in-field and out-of-field recurrence) should be considered as detrimental for IMRT. However, some meaningful strengths of IMRT in combination with IF-RT have to be weighed against its potential adverse effects on tumor control in elective nodal regions. As we could demonstrate, IMRT opens the door to a significant dose-escalation or alternatively embodies the benefit of substantial sparing of normal tissues at an equal total dose to that of 3D-CRT.

To our knowledge this is the first study examining the dose distribution in elective lymph node stations adjacent to the CTV in the context of IF-IMRT in NSCLC. Several planning studies examined the potential benefits of incidental nodal irradiation of uninvolved lymph node regions when using 3D-CRT [7, 8, 20, 21]. Even with PET-based IF-RT of stage III NSCLC, rates of out-of-field nodal recurrences can be as high as 10 % and therefore cannot be considered as insignificant [22]. It is controversial if these recurrences can be attributed either to the small *a priori* risk of microscopic involvement in elective nodal regions or the benefits of incidental nodal irradiation. Yet, thorough dosimetric analyses of isolated out-of-field nodal failures indicate a dose–response relationship, and a critical cutoff may be in the range of 40–50 Gy [7, 21]. Depending on the total burden of subclinical disease a wide range of therapeutic total doses can be assumed [23]. For a collective of patients with node-negative head and neck cancer a reduction of neck relapses by more than 90 % with total doses of 50 Gy and by less than 50 % with less than 30 Gy (2 Gy single fraction) was reported [24]. In that context it has to be stressed that the calculation of the presented TCP values is inaccurate inasmuch as neither the ‘true’ load of subclinical disease nor the ‘true’ tumoricidal doses remain known for the average patient. Nevertheless, the use of somewhat arbitrary radiobiologic parameters for TCP-estimation still provides some guidance when comparing individual dose disbributions. Also, the TCP-values should be regarded as complementary to the by far more objective EUD-concept.

As the use of IMRT has been widely adopted for inoperable stage III NSCLC our results underline the importance of keeping track of out-of-field recurrences at times when highly conformal dose distributions and tight margins around the CTV are applied in the era of image-guided radiotherapy.

## Conclusions

Incidental irradiation of out-of-field lymph node stations in NSCLC may be beneficial with respect to control of microscopic lymph node involvement. With IMRT, smaller doses are deposited in lymph node stations adjacent to the CTV due to its higher conformality as compared to 3D-conformal radiotherapy. The planning study presented here shows a moderate yet statistically significant reduction in hypothetical tumor control probability for microscopic involvement in out-of-field lymph node stations for IMRT vs. 3D-CRT at equal dose levels. This potential drawback may be outweighed by the superiority of IMRT in sparing organs at risk and by its potential to achieve higher total doses in the GTV. Yet, accurate – i.e. FDG-PET based – lymph node staging seems to be an important prerequisite for IMRT.
